# Moving towards optimized treatment for children and adolescents with juvenile idiopathic arthritis in sustained remission randomized to continue stable treatment, methotrexate withdrawal or tumor necrosis factor inhibitor withdrawal: study protocol for the Norwegian multi-center MOVE-JIA trial

**DOI:** 10.1186/s13063-026-09539-0

**Published:** 2026-02-17

**Authors:** Siri Opsahl Hetlevik, Vibke Lilleby, Maiju Pesonen, Ellen Nordal, Marite Rygg, Cathrine Austad, Maria Karolina Jonsson, Maria Bilstad, Hege Kilander Høiberg, Nina Krafft Sande, Berit Flatø, Siri Lillegraven, Espen A. Haavardsholm, Athimalaipet V. Ramanan, Øyvind Molberg, Pernille Bøyesen, Anna-Birgitte Aga

**Affiliations:** 1 Department of Rheumatology, Oslo University Hospital, Oslo, Norway; 2 Oslo Centre for Biostatistics and Epidemiology, Oslo University Hospital, Oslo, Norway; 3 Department of Clinical Medicine, The Arctic University of Norway (UiT), Tromsø, Norway; 4 Department of Pediatrics, University Hospital of North Norway (UNN), Tromsø, Norway; 5 Department of Clinical and Molecular Medicine (IKOM), Norwegian University of Science and Technology (NTNU), Trondheim, Norway; 6 Department of Pediatrics, St. Olavs Hospital, Trondheim, Norway; 7 Department of Rheumatology, Vestre Viken Hospital Trust, Drammen, Norway; 8 Children and Youth Clinic, Haukeland University Hospital, Bergen, Norway; 9 Department of Pediatrics, Stavanger University Hospital, Stavanger, Norway; 10 Department of Rheumatology, Sørlandet Hospital Trust, Kristiansand, Norway; 11 Faculty of Medicine, University of Oslo, Oslo, Norway; 12 Diakonhjemmet Hospital, Center for Treatment of Rheumatic and Musculoskeletal Diseases (REMEDY), Oslo, Norway; 13 Bristol Royal Hospital for Children and Translational Health Sciences, University of Bristol, Bristol, UK

**Keywords:** Maintenance treatment, Medication withdrawal, JIA, TNF-inhibitors, Methotrexate

## Abstract

**Background:**

Juvenile idiopathic arthritis (JIA) used to be a joint-destroying disease, but thanks to modern treatment strategies and medications, many patients with JIA today reach inactive disease. However, once disease remission is achieved, there is a lack of knowledge and recommendations regarding maintenance therapy. Drug-free remission is the ultimate goal in JIA, but withdrawal of medications increases the risk of disease flare. Clinical approaches vary widely, underscoring a need for knowledge about maintenance treatment strategies that allow for safe tapering and withdrawal of medications in JIA patients in sustained remission. The MOVE-JIA study is a randomized, controlled trial with the primary objective to compare the effect of two different treatment withdrawal strategies, to a stable dose of methotrexate (MTX) and tumor necrosis factor inhibitors (TNFi), based on the risk of flares in children and adolescents with JIA with sustained inactive disease. A key secondary objective is the proportion of children with disease flare compared between the two withdrawal groups.

**Methods:**

In this investigator-initiated multicenter, randomized, 3-grouped, parallel, open-label, noninferiority trial, treating physicians at seven Norwegian pediatric rheumatology hospital centers will include 150 patients with JIA. Key eligibility criteria are as follows: Fulfilment of the International League of Associations for Rheumatology (ILAR) classification criteria for non-systemic JIA, inactive disease for ≥ 12 months documented at a minimum of 2 consecutive visits, and no active uveitis for ≥ 24 months under treatment with stable doses of MTX and TNFi.

They will be randomized in a 1:1:1 ratio to (A) stable treatment, (B) methotrexate withdrawal, or (C) TNFi withdrawal. Randomization will be stratified for JIA subtype and study center. For patients in group B and C who are still in remission after 12 months, a new randomization will be performed for complete medication withdrawal for the next 12 months. After 24 months, medication adjustments will be done with shared decision-making. The primary endpoint is the rate of disease flare compared between the drug withdrawal groups and the stable treatment group between baseline and 12 months follow-up. The key secondary endpoint is the proportion with disease flare compared between the two withdrawal groups. Incidence and severity of adverse events will be monitored.

**Discussion:**

The results from the MOVE-JIA trial will present an evidence-based treatment strategy for JIA patients with inactive disease. The trial will also give us knowledge about regaining disease remission after flares and possibilities of drug-free remission. All outcomes from the trial will provide a scientific basis for optimized JIA care and result in new treatment recommendations.

**Trial registration:**

EU CT 2024–512017–12–00. Registered on October 24th, 2024; ClinicalTrials.gov NCT06653634. Registered on October 24th, 2024.

URL: Study Details | NCT06653634 | Optimizing Treatment for Patients With Juvenile Idiopathic Arthritis in Sustained Remission: The MOVE–JIA Trial | ClinicalTrials.gov.

Date of first recruitment: October 24th, 2024.

## Administrative information

Note: the numbers in curly brackets in this protocol refer to SPIRIT checklist item numbers. The order of the items has been modified to group similar items (see http://www.equator-network.org/reporting-guidelines/spirit-2013-statement-defining-standard-protocol-items-for-clinical-trials/).

**Table:** 

Title {1}	Moving towards optimized treatment for children and adolescents with juvenile idiopathic arthritis in sustained remission randomized to continue stable treatment, methotrexate withdrawal or tumor necrosis factor inhibitor withdrawal: study protocol for the Norwegian multi-center randomized controlled MOVE-JIA trial?
Trial registration {2a and 2b}.	EU CT 2024–512017-12–00, October 24th, 2024, (Search for clinical trials—EMA) NCT06653634, October 24 2024, (Study Details | Optimizing Treatment for Patients with Juvenile Idiopathic Arthritis in Sustained Remission: the MOVE-JIA Trial | ClinicalTrials.gov)
Protocol version {3}	Protocol version 1.2, 06.05.2025
Funding {4}	This study is fully funded by external sources and has undergone full external peer review. The study has received funding from the South-Eastern Norway Regional Health authority, The Research Council of Norway, Norwegian Women’s Public Health Association, and Center for Treatment of Rheumatic and Musculoskeletal Diseases (REMEDY).
Author details {5a}	Siri Opsahl Hetlevik1, Vibke Lilleby1, Maiju Pesonen2, Ellen Nordal3, 4, Marite Rygg5, 6, Cathrine Austad7, Maria Karolina Jonsson8, Maria Bilstad9, Hege Kilander Høiberg10, Nina Krafft Sande1, Berit Flatø1,11, Siri Lillegraven12, Espen A. Haavardsholm11,12, Athimalaipet V. Ramanan13, Øyvind Molberg 1,11, Pernille Bøyesen*1, Anna-Birgitte Aga*1 1Oslo University Hospital, Department of Rheumatology, Oslo, Norway, 2 Oslo University Hospital, Oslo Centre for Biostatistics and Epidemiology, Oslo, Norway, 3The Arctic University of Norway (UiT), Department of Clinical Medicine, Tromsø, Norway, 4University Hospital of North Norway (UNN), Department of Pediatrics, Tromsø, Norway, 5Norwegian University of Science and Technology (NTNU), Department of Clinical and Molecular Medicine (IKOM), Trondheim, Norway, 6St. Olavs Hospital, Department of Pediatrics, Trondheim, Norway, 7Vestre Viken Hospital Trust, Department of Rheumatology, Drammen, Norway, 8Haukeland University Hospital, Children and Youth Clinic, Bergen, Norway, 9Stavanger University Hospital, Department of Pediatrics, Stavanger, Norway, 10Sørlandet Hospital Trust, Department of Rheumatology, Kristiansand, Norway, 11 University of Oslo, Faculty of Medicine, Oslo, Norway, 12Diakonhjemmet Hospital, Center for Treatment of Rheumatic and Musculoskeletal Diseases (REMEDY), Oslo, Norway 13 Bristol Royal Hospital for Children and Translational Health Sciences, University of Bristol, Bristol *Drs Bøyesen and Aga contributed equally to the article
Name and contact information for the trial sponsor {5b}	Department of Rheumatology Division of surgery, Inflammatory diseases and Transplantation Legal registered address: Oslo University Hospital Contact person: Jan Cezary Sitek Postbox 4950 Nydalen, 0424 OSLO E-mail: jsitek@ous-hf.no
Role of sponsor {5c}	This study is initiated and designed by researchers. The study sponsor and the funders have no role or authority over study design, collection, management, analysis, and interpretation of data; writing of the report, or the decision to submit the report for publication.

## Introduction

### Background and rationale {6a}

MOVE-JIA is a randomized controlled trial (RCT), where we will develop evidence-based maintenance and treatment tapering strategies in patients with juvenile idiopathic arthritis (JIA) in sustained remission.

JIA encompasses several distinct entities that begin in childhood or adolescence, characterized by the onset of joint inflammation. JIA is estimated to affect 1 in 1000 children and adolescents from 1 to 16 years of age. The prognosis worsens with increased number of involved joints, and the disease often persists into adulthood [1]. JIA carries a risk of disability and significantly impacts school attendance, social life, and leisure activities.

Advances in treatment strategies including biologic and conventional synthetic disease-modifying anti-rheumatic drugs (DMARDs), mainly methotrexate (MTX) and tumor necrosis factor inhibitors (TNFi), have improved outcomes for children and adolescents with JIA. Sustained inactive disease with no signs or symptoms of inflammation is now an obtainable goal [2]. However, once remission is achieved, there is limited evidence to guide decisions regarding maintenance therapy and treatment tapering.

There are no available international recommendations on maintenance therapy and medication withdrawal, but it seems that flares are common after cessation of treatment, occurring from 10 weeks and up to several years thereafter remission [3–5]. One RCT conducted by the Pediatric Rheumatology International Trials Organization (PRINTO) compared discontinuation of MTX after 6 or 12 months of remission and reported a relapse rate of 57% and 56% in the two groups (odds ratio 1.02; 95% CI 0.82 to 1.27) within 24 months [6]. Observational data from The Research in Arthritis in Canadian Children emphasizing Outcomes (ReACCh-Out) cohort showed a probability of flare of 32% (95% CI 0.28–0.36) within the first year after treatment discontinuation in patients that had reached inactive disease [4]. For TNFi, no RCTs on maintenance treatment and medication withdrawal have been conducted, but according to available literature reported flare rates vary from 30% to as high as 60–80% within the first 12 months after treatment withdrawal [7, 8].

Data are also limited regarding the clinical course following disease flare after treatment withdrawal. One study found that 54% of flares in patients after DMARD withdrawal involved at least one joint not previously affected, illustrating a possible risk for a more severe disease course after each flare [5].

Conclusive evidence on the management of JIA in remission is requested. To our knowledge, no studies have focused on strategies to step down combination therapy in these patients.

#### Benefits and potential harms of maintenance and withdrawal strategies

Across all treatment strategies, disease flares represent a potential harm, as flares are associated with pain, functional impairment, reduced participation in school and activities, and the risk of cumulative joint damage [4]. Continued combination therapy with TNFi and MTX minimizes the risk of disease flares and maintain sustained control of inflammation, thereby limiting the risk of structural joint-damage and functional impairment [9]. However, continued therapy is not a guarantee for sustained remission and disease flares do occur [10]. Prolonged exposure to combination therapy is also associated with medication-related side effects, toxicities, treatment burdens, and increased healthcare costs [11–13].

Withdrawal of one component of combination therapy may reduce medication exposure and treatment burden. Discontinuation of MTX with continuation of TNFi may be preferred by the children and adolescents due to side effects of MTX and may affect adherence [11]. TNFi treatment is shown to protect better against joint damage compared to MTX monotherapy in severe JIA ADDIN EN.CITE [9], but concerns remain about long-term side effects and the risk of potential development of anti-drug antibodies in the absence of concomitant MTX. Across studies, flare rates following MTX withdrawal are reported to range from 30 to 50% [4, 6].

Discontinuation of TNFi with continuation of MTX may reduce exposure to biologic therapy and lower treatment costs. MTX monotherapy may be sufficient to maintain remission in some patients, although available data suggest that the risk of flare following TNFi withdrawal may be as high as 60% [8]. In addition, MTX monotherapy may offer less protection against structural joint damage in patients with more severe disease [9, 14]

Evidence regarding the ability to regain remission after flare is limited and the long-term implications of treatment withdrawal remain uncertain.

#### Rationale for the MOVE-JIA trial

Current clinical practice regarding maintenance therapy in JIA patients who have reached inactive disease under treatment with methotrexate and TNFi varies widely between centers and countries, reflecting the lack of international recommendations.

The MOVE-JIA trial is designed to address this evidence gap by comparing continued combination therapy with two withdrawal strategies in this patient population. The results will provide a scientific basis for optimized JIA care in all countries where biologic therapy is available.

## Objectives {7}

The primary objective of the MOVE-JIA trial is to compare the effect of two different treatment withdrawal strategies, to a stable dose of MTX and TNFi, based on the risk of flares in children and adolescents with JIA with sustained inactive disease. The key secondary objective is the proportion of children with disease flare compared between the two withdrawal groups. Other important aims are to determine time to flare and time to regain remission after flare, to compare changes in disease activity in the different treatment groups, and to evaluate overall safety.

## Trial design {8}

The MOVE-JIA study is an investigator-initiated multicenter, randomized, 3-grouped (allocation 1:1:1), parallel, open-label, noninferiority trial designed to compare the proportion of study participants with disease flares in the different treatment groups. Oslo University Hospital is study sponsor. Patients will be followed for 3 years through three study periods, where the primary endpoint will be assessed after study period 1 (12 months).

## Methods: Participants, interventions, and outcomes

### Study setting {9}

The study is initiated and coordinated from the Department of Rheumatology, Oslo University hospital, Oslo, Norway, and conducted at seven different study sites (five university hospitals and two regional/community hospitals) across Norway treating children and adolescents with JIA. These study sites cover most of all JIA patients treated in Norway for the age groups included in this study. For detailed information on study sites, see ClinicalTrials.gov.

### Eligibility criteria {10}

Patients are eligible to enroll in the study if they meet the following criteria at screening and at baseline (Table 1).

**Table 1:** Inclusion and exclusion criteria

Inclusion criteria
Participants are eligible to be included in the study only if all the following criteria apply:
Age 1. Participant must be 2–18 years of age at the time of signing the informed consent
Type of participant and disease characteristics 2. Fulfilment of the International League of Associations for Rheumatology (ILAR) classification criteria for non-systemic JIA 3. Inactive disease for ≥ 12 months documented at a minimum of 2 consecutive visits and documented inactive disease according to Wallace 2011 criteria at inclusion, and no active uveitis for ≥ 24 months 4. Stable treatment with MTX and TNFi for ≥ 6 months. Weight adjustments permitted
Informed consent 5. Capable of giving signed informed consent which includes compliance with the requirements and restrictions listed in the informed consent form
Contraceptive use by men and women 6. Male participants: No measures necessary 7. Female participants: Contraception guidance for women of childbearing potential (WOCP)
Exclusion criteria
Participants are excluded from the study if any of the following criteria apply:
Medical conditions 1. Chronic widespread pain syndrome 2. Major comorbidity including uncontrolled infectious, neurological or mental disease, malignant disease, severe heart failure, severe renal failure, active ulcus ventriculi, and uncontrolled diabetes mellitus
Prior/concomitant therapy 3. Use of oral, intra-articular, intramuscular, or intravenous corticosteroids due to JIA less than 12 months prior to randomization
Prior/concurrent clinical study experience 4. Participating in an ongoing clinical randomized study
Other exclusion criteria 5. Drug/alcohol abuse which hampers adherence to the study protocol as based on the investigators’ judgement 6. Language barriers that hamper adherence to the study protocol 7. Pregnancy or breastfeeding 8. Any condition that in the view of the investigator would suggest that the patient is unable to comply with the study protocol and procedures 9. Unwillingness to use safe contraception for sexually active WOCP

### Who will take informed consent? {26a}

The local study personnel at each site will obtain informed consent from the legal guardians and patients, as appropriate at screening.

### Additional consent provisions for collection and use of participant data and biological specimens {26b}

The consent includes permission to collect and analyze data and blood samples, including biobanking.

## Interventions

### Explanation for the choice of comparators {6b}

MTX and TNFi are the most commonly prescribed drugs to treat JIA. Combination therapy with MTX and TNFi is used for patients with more severe disease, either because they fail to achieve remission on monotherapy with MTX or because they have poor prognostic factors (such as positive rheumatoid factor or anti-citrullinated protein, radiographic joint damage, joint distribution of active joints, or otherwise high disease activity). Nevertheless, after disease activity is controlled and remission is attained, it remains uncertain whether continuing a combination of two immunosuppressants—each known to be effective in JIA—is required to maintain inactive disease. The study is designed to compare continued stable combination therapy with two medication tapering strategies in a real-world clinical context. The use of placebo is ethically problematic in pediatric populations, particularly when effective therapies are available, and is therefore not appropriate in this setting.

### Intervention description {11a}

**Fig. 1:**
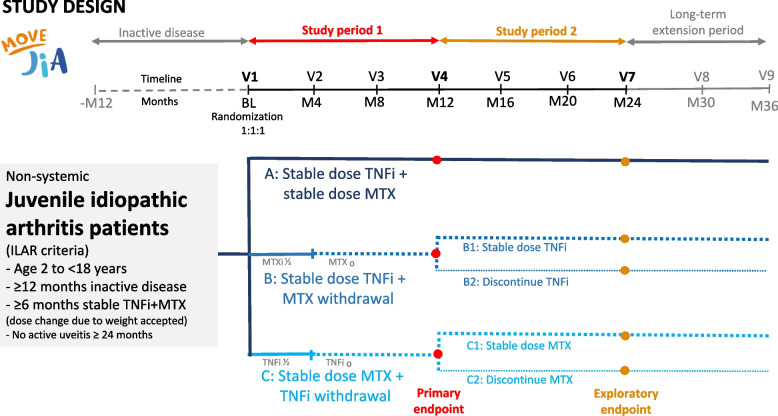
Study design. JIA, juvenile idiopathic arthritis; ILAR, international league against rheumatism; TNFi, tumor necrosis factor inhibitor; MTX, methotrexate; V, visit

In study period 1, children and adolescents with JIA with sustained inactive disease will be randomized in a 1:1:1 ratio to either (A) continued stable TNFi + MTX, (B) stable TNFi and stepwise MTX withdrawal, or (C) stable MTX and stepwise TNFi withdrawal (Fig. 1). Randomization is stratified for JIA subtype and study center.

**Fig. 2:**
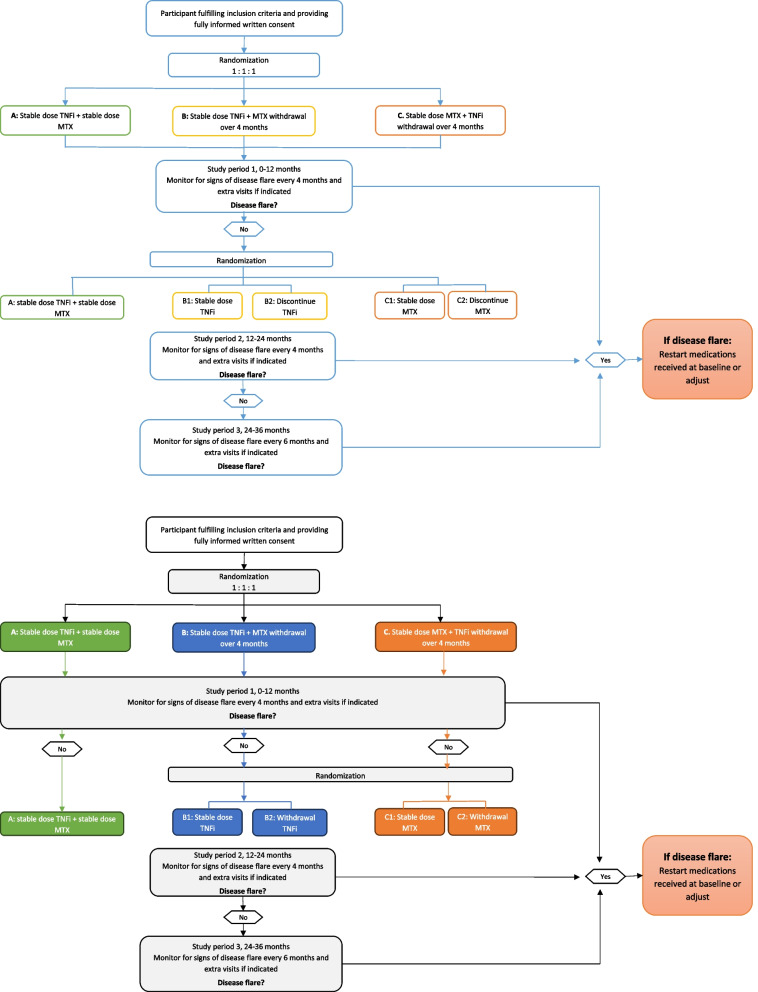

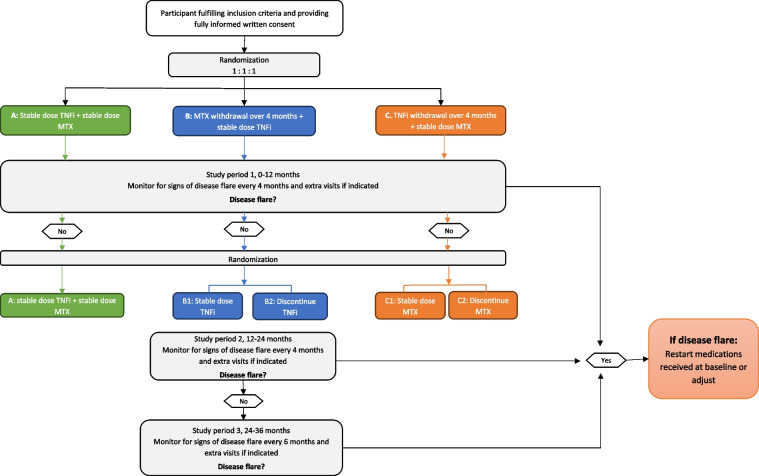
Patient flow

In study period 2, study participants in groups B and C who have not experienced a disease flare from baseline to 12 months will be randomized 1:1 to either B1: stable dose TNFi or B2: stepwise TNFi withdrawal; or C1: stable dose MTX or C2: stepwise MTX withdrawal. In study period 3, patients will be followed, and treatment adjustments will be performed in shared decision making. In case of disease flare, medications received at baseline will be restarted or adjusted as appropriate (Fig. 2a and b, patient flow).

Participants and treating clinicians are not blinded to the group allocation due to ethical considerations, but the statistician that will perform the primary outcome analyses is blinded.

### Criteria for discontinuing or modifying allocated interventions {11b}

In case of disease flare in group B or C, medications used at baseline should be restarted, and patients with flare in group A will be treated according to current recommendations. If a patient develops infections with fever or needs antibiotics, the medications should be paused as per clinical practice. The same applies for side effects such as significant laboratory abnormalities, pregnancy, or other adverse events. Participants can resume the prescribed medications as soon as the investigator deems it safe.

### Strategies to improve adherence to interventions {11c}

Patients eligible for this study are used to taking prescribed medicines regularly and are familiar with the medications and their side effects. At every visit, we will address compliance and register this in the electronic case report file (eCRF).

### Relevant concomitant care permitted or prohibited during the trial {11d}

There is no concomitant care prohibited during the trial. All concomitant care is registered.

### Provisions for post-trial care {30}

All patients will be followed for 36 months in the study. In case of disease flare, medications received at baseline will be restarted with adjustments if necessary to achieve inactive disease. After the study, patients will be taken care of in regular clinical practice. No compensation will be provided for the participants.

### Outcomes {12}

#### Primary outcome

The primary outcome of this study is the proportion of study participants with a disease flare (yes or no) during the first 12 months of follow-up compared between each of the two drug withdrawal groups and the stable treatment group. Disease flare is defined as a combination of the following:

A clinically significant increase in Juvenile Arthritis Disease Activity Score 27 (JADAS-27) ≥ 1.7 from baseline AND active joints ≥ 1 (swollen, or tender + limited range of motion) OR consensus between treating physician and participant/parents that a clinically significant flare has occurred with the need for intensification of DMARD treatment.

The JADAS is a validated composite outcome measure of JIA disease activity, calculated as a sum of scores from its four components: Physician global assessment of disease activity on a visual analogue scale from 0 to 100 mm (higher scores indicating higher disease activity), parent/patient global assessment of well-being on a visual analogue scale from 0 to 100 mm (higher scores indicating less global well-being), active joint count of 27 joints, and erythrocyte sedimentation rate (ESR mm (hr) [15]. Disease flare based on consensus between treating physician and patient/parent (yes/no) can include uveitis flare, arthritis on imaging, psoriasis, inflammatory back pain, enthesitis, or other factors.

#### Key secondary outcome

The key secondary outcome is the proportion of study participants with a disease flare (disease flare is defined above) during the first 12 months’ follow-up compared between the two drug withdrawal groups.

#### Secondary outcomes

Secondary outcomes include time to flare and time to regain inactive disease (according to the Wallace 2011 criteria) after flare (as defined above), changes in validated measures of disease activity and reports of adverse events, serious adverse events, and suspected unexpected serious adverse reactions [16, 17]. The Wallace criteria for inactive disease require no active arthritis, no active uveitis, no systemic features, normal ESR or CRP, physician global assessment of disease activity on a visual analogue scale = 0, and morning stiffness < 15 min [18].

### Participant timeline {13}

All patients will be followed with data collection every 4 months in study period 1 and 2, and with extra visits if indicated. At visit 0, baseline measurements will be captured before randomization. In study period 3, patients will be followed every 6 months with extra visits if indicated (Table 2, schedule of activities (SOA) and Fig. 2, patient flow).

**Table 2:** Schedule of activities

Study visits (months)	0	4	8	12	16	20	24	30	36	EV
History										
Informed consent/inclusion and exclusion criteria	x									
JIA classification and history/medical history	x									
Comorbidity/medication/adverse events	x	x	x	x	x	x	X	x	x	x
Randomization	x			x						
Clinical assessments										
Joint examination/core set variables for JIA	x	x	x	x	x	x	X	x	x	x
Physician overall assessment of disease activity	x	x	x	x	x	x	X	x	x	x
Disease flare yes/no	x	x	x	x	x	x	X	x	x	x
Patient reported variables										
Pain, fatigue, patient global visual analogue scale	x	x	x	x	x	x	X	x	x	x
Physical function, disease specific health-related quality of life, treatment satisfaction, physical activity, and school absenteeism	x	x	x	x	x	x	X	x	x	x
Psychological health and general quality of life	x			x			X		x	x
Biochemical examinations and biobank										
JIA markers: anti-nuclear antibodies, rheumatoid factor, anti-citrullinated protein antibodies and HLA-B27	x	x	x	x	x	x	X	x	x	x
Inflammatory markers: erythrocyte sedimentation rate/C-reactive protein	x	x	x	x	x	x	X	x	x	x
Biobanking: full blood	x	x	x	x	(x)	(x)	X	(x)	x	x
EV, extra visits; JIA, juvenile idiopathic arthritis; (x), biobanking optional										

Patients will see a pediatric rheumatologist at every visit with review of health status, disease-related concerns, blood tests, joint examination, and recording of medication use and adverse events as well as patient-reported outcomes (PROMs). Patients who are unable to attend a visit will be offered a rescheduled visit. All patients will be given contact information to the department or the study nurse in case they suspect a disease flare or have other questions. If participants experience a potential disease flare, an extra visit will be arranged within 2 weeks to allow for examination of disease status. If a disease flare occurs, the participant will return to the full dose study medication (baseline treatment) or treatment adjusted according to current recommendations.

Research data are collected at scheduled visits during the study.

### Sample size {14}

The primary objective is to assess the non-inferiority of each of the drug withdrawal groups B and C to control group A in terms of disease flare (yes/no) during the 12-month follow-up. Due to two primary comparisons, a Bonferroni correction is applied to account for multiple testing, resulting in a significance level of 2.5%. Further, non-inferiority is assessed by comparing the upper bound of the 97.5% confidence interval of the risk difference to the pre-specified 20% non-inferiority margin. Thus, a significance level of 1.25% is used when determining the required sample size.

Available data suggest that the rate of disease flare when continuing stable treatment (group A) is approximately 10%. Due to the non-inferiority set-up, we assume that the risk of disease flare is equal in all three groups. Using a non-inferiority margin of 20%, power of 80%, and significance level of 1.25%, we estimate that randomization of 43 patients per group would be required. Assuming a 15% drop-out rate, a total of 150 participants will be randomized.

### Recruitment {15}

Patients will be recruited from clinical practice, through chart reviews, and from the ongoing open extension of the MyJIA trial (ClinicalTrials.gov, NCT04614311).

## Assignment of interventions: allocation

### Sequence generation {16a}

Randomization will be performed using a computer-generated random sequence, set up by the clinical trial unit at OUH, Norway, and performed by the study statistician. Assignment to treatment group will be performed through the eCRF Viedoc™, Uppsala, Sweden. Stratification for JIA subtype and study center will be performed in the eCRF.

### Concealment mechanism {16b}

Randomization will be performed in the eCRF. The study is not blinded, and it will be clearly stated in the eCRF what treatment the patient is randomized to.

### Implementation {16c}

The allocation sequence will be generated by a computer-based block randomization stratified by study center and JIA subtype, integrated in the electronic data capture software (Viedoc™). Study personnel will enroll participants and enter the information needed in the eCRF before the randomization (inclusion and exclusion criteria, JIA subtype, and registration of fulfillment of Wallace clinical inactive disease 2011 criteria at baseline) [18].

## Assignment of interventions: Blinding

### Who will be blinded {17a}

Not applicable; this is an open-label study.

### Procedure for unblinding if needed {17b}

Not applicable; this is an open-label study.

## Data collection and management

### Plans for assessment and collection of outcomes {18a}

The primary outcome will be assessed on every study visit and extra visits. For detailed information about data collected on each visit, see Table 2 (SOA). Data are reported in the eCRF. PROMs will be collected directly in the eCRF using tablets. All study personnel will undergo training before trial initiation and repeated annually during data collection.

### Plans to promote participant retention and complete follow-up {18b}

Given the eligibility criteria, all participants in this study have been under long-term follow-up from before study start and thus have an established relationship with the clinic. Participants will be given a direct number to the study nurse or clinic for contact regarding the study, the medications, side effects, or if they suspect a disease flare. If a participant chooses to leave the study, he or she will be scheduled for an early discontinuation visit and thereafter be followed in clinical practice.

### Data management {19}

Data are reported directly in the eCRF, Viedoc, which requires two-factor authentication. The investigator must permit study-related monitoring, audits, ethics committee review, and regulatory agency inspections and provide direct access to source documents. Records and documents, including signed informed consent forms, must be retained by the investigator for 25 years after study completion unless local regulations or institutional policies require a longer retention period. No records may be destroyed during the retention period without the written approval of the sponsor. No records may be transferred to another location or party without written notification to the sponsor. Independent data monitoring is organized by the Clinical Trial Unit, Oslo University Hospital.

### Confidentiality {27}

Informed consent signed by both the participants’ legal guardians is required prior to any data collection in the eCRF. Patient confidentiality will be maintained according to the data privacy statement in the informed consent form.

### Plans for collection, laboratory evaluation, and storage of biological specimens for genetic or molecular analysis in this trial/future use {33}

Routine laboratory tests will be analyzed locally at each participating site. Assessment of drug and anti-drug antibody levels will be analyzed at the Department of Biochemistry at Oslo University Hospital, Radiumhospitalet, Norway. Biobanking of blood will be collected at every visit. Biobank samples will be stored locally in a freezer at − 70 to − 80 °C until all biobank material is collected. At the end of the study, biobank material will be shipped to the sponsor according to procedure.

## Statistical methods

### Statistical methods for primary and secondary outcomes {20a}

The sample size was determined by using R-function *ssc_propcomp* (non-inferiority hypothesis) in *SampleSize4ClinicalTrials* -package (R Foundation for Statistical Computin, Vienna, Austria). R or statistical software for data science (STATA) will be used to analyze the results of the study.

The main analyses will be performed according to a predefined Statistical Analysis Plan (SAP) when all participants have concluded the study, all data have been entered, verified, and validated, and the database has been locked. The SAP can be provided upon request to the authors. The intention-to-treat (ITT) set will include all patients randomly assigned to a treatment group irrespective of post-randomization occurrences. The per-protocol analysis set (PPS) will include all randomized patients meeting the study entry criteria who followed the study protocol with no major protocol deviations. The primary analyses will be performed based on the PPS. Due to the non-inferiority setting, a per-protocol approach was chosen to assess the effect among participants who adhered to the protocol to avoid bias toward no treatment difference, thereby reflecting the effect under optimal implementation, while the ITT set will be used for sensitivity analysis. The safety analyses will be performed using the ITT set.

Baseline characteristics will be summarized descriptively by treatment group using mean and standard deviation or median and interquartile range for continuous variables, and counts and percentages for categorical variables.

The primary objective of the trial is to assess the non-inferiority of each of the drug withdrawal groups B and C to control group A. The two primary hypotheses are the following:

1. Group B (withdrawal of MTX, stable TNFi) is non-inferior to group A (stable MTX and TNFi), i.e., the difference between the groups in the proportions of study participants having disease flares during the 12-month follow-up period is less than or equal to the pre-specified non-inferiority margin of 20%.
2. Group C (withdrawal of TNFi, stable MTX) is non-inferior to group A (stable MTX and TNFi).

As two primary non-inferiority tests are performed simultaneously, a Bonferroni correction will be applied, and primary endpoints tested at the 0.025 level to control the overall probability of Type I error. The non-inferiority of groups B and C to group A will be evaluated by estimating a risk difference and its 97.5% confidence interval from the logistic regression model with disease flare as a binary outcome and treatment group and stratification variables (study site and JIA course (persistent oligoarticular JIA versus other non-systemic JIA categories)) as covariates. The upper bound of this confidence interval will be compared to the non-inferiority margin of 20%. Non-inferiority is claimed if the primary null hypothesis is rejected at the significance level of 0.0125 (one-sided), that is, if the upper limit of the 97.5% two-sided confidence interval for the treatment difference is less than 20%. In case the primary null hypothesis (1) or (2) is not rejected (group B/C is inferior to group A), the superiority of group A to group B/C will be examined (key secondary).

Key secondary objective is to compare the disease flare rates during the first 12 months follow-up in two drug withdrawal groups B and C. The key secondary hypothesis is that the risk of disease flare during the first 12 months of follow-up is equal in arm B (withdrawal of MTX with stable TNFi) and arm C (withdrawal of TNFi with stable MTX). The comparison will be performed as a two-sided superiority test by estimating a risk difference and corresponding 95% confidence interval from the logistic regression model with disease flare as a binary outcome and treatment group and stratification variables (study site and JIA course (persistent oligoarticular JIA versus other non-systemic JIA categories)) as covariates. Based on the results, the direction and the clinical relevance of the difference may be examined.

The results of the trial will be reported according to consolidated standards of reporting trials (CONSORT).

### Interim analyses {21b}

No interim analyses will be performed in this study.

### Methods for additional analyses (e.g. subgroup analyses) {20b}

Methods for additional analyses will be described in the SAP.

### Methods in analysis to handle protocol non-adherence and any statistical methods to handle missing data {20c}

Missing data and reason for the missingness will be presented. Missing data for the primary endpoint will be imputed with a negative outcome (flare). Comparisons of missing data between treatment groups and robustness analyses including missing data imputation will also be performed according to the SAP.

### Plans to give access to the full protocol, participant level-data and statistical code {31c}

MOVE-JIA is registered on clinicaltrials.gov and on Clinical Trials in the European Union (CTIS). The full trial protocol (version 1.2, 06.05.2025) and statistical code are available from the corresponding author upon request. At present, data sharing is not applicable.

## Oversight and monitoring

### Composition of the coordinating centre and trial steering committee {5d}

The Department for Rheumatology at Oslo University Hospital (OUH) is the coordinating center for the project. The Pediatric Rheumatology Research Group has performed numerous open studies on disease outcomes and clinical genetic predictors in JIA patients, and a recently completed randomized controlled trial on JIA treatment (the MyJIA trial, ClinicalTrials.gov: NCT04614311). The study has been planned and will be performed in collaboration with the Norwegian Clinical Research Infrastructure Network (NorCrin), the center for treatment of rheumatic and musculoskeletal diseases (REMEDY), and the research group works closely with the Clinical Trial Unit (CTU) at OUH. The CTU offers advising in GCP, data management, monitoring, statistics, and more. A steering committee has been appointed, including two members independent of the study.

### Composition of the data monitoring committee, its role and reporting structure {21a}

There will not be a data monitoring committee for this study.

### Adverse event reporting and harms {22}

Adverse events (AE) will be collected at every study visit from the start of the study until the last follow-up visit. An adverse event will be considered serious (SAE) if it results in any of the following: death, a life-threatening adverse event, in-patient hospitalization or prolongation of hospitalization, disability or permanent damage, or a congenital anomaly/birth defect. All SAEs will be recorded and reported to the sponsor or designee immediately and under no circumstances should this exceed 24 h. If a SAE occurs, the investigator will determine if it is related to the study medication based on clinical judgement. The degree of certainty about the association will be certain, probable, possible, or unlikely. The medical monitor and the trial sponsor will be notified automatically through the eCRF.

### Frequency and plans for auditing trial conduct {23}

The coordinating center will remotely evaluate the quality of trial activities if indicated. In-person site visits with the monitor will be conducted at regular intervals, estimated once yearly during the recruitment period and one visit after the last patient’s last visit. As Oslo University Hospital will recruit the majority of patients, monitoring will be conducted twice a year during the recruitment period at this center.

### Plans for communicating important protocol amendments to relevant parties (e.g. trial participants, ethical committees) {25}

All protocol amendments must be approved by the ethics committee and the Norwegian Medical Products Agency through the CTIS system before all study centers will receive the updated version of the protocol. Regular newsletters will be sent to all study personnel and user representatives.

## Dissemination plans {31a}

Communication of results from the trial will be based on peer-reviewed publications, websites, and in collaboration with user societies. Multiple abstracts will be submitted to national and international congresses.

## Discussion

The MOVE-JIA trial is the first randomized controlled trial comparing stable combination therapy with MTX and TNFi to treatment withdrawal strategies for children and adolescents with JIA in remission. While there is limited evidence on the effect of discontinuing therapy, tapering or withdrawing medications is common practice in pediatric rheumatology. This trial aims to address this knowledge gap and provide much-needed evidence to guide treatment decisions. The results from the trial will directly translate into evidence-based clinical decision making for JIA patients with sustained inactive disease. All potential outcomes of the study may influence clinical practice and treatment recommendations and guidelines.

The main objective is to determine if the drug withdrawal groups are non-inferior to stable treatment with TNFi and MTX in children and adolescents with JIA in sustained inactive disease, i.e., if withdrawal of MTX and stable treatment with TNFi is non-inferior to stable combination therapy, and if stable treatment with MTX and withdrawal of TNFi is non-inferior to stable combination treatment. Should either of the treatment withdrawal strategies demonstrate non-inferiority, this would have important implications for clinical practice with a shift in mode/pattern and extent of treatment tapering in more patients. Key secondary objective is to assess the risk of disease flares between the two different withdrawal strategies. Should TNFi monotherapy prove non-inferior to continued combination therapy and superior to MTX monotherapy, this could support simplifying long-term treatment to TNFi alone, potentially reducing the burden of care and side effects for patients. Such evidence would inform updated treatment guidelines and support more personalized treatment decisions based on individual tolerability.

In addition to flare rates, the study will provide valuable insights into the ability to regain remission following a disease flare. Understanding how readily patients can achieve inactive disease after a flare, and how long this takes, will help clinicians balance the benefits of tapering with the potential risks. The study will also explore the risk of developing anti-drug antibodies in patients undergoing treatment changes, and the possibility of achieving drug-free remission, a long-term goal for many patients and caregivers. The results may contribute to better evidence-based information for shared decision-making, ultimately improving long-term health, functional outcomes, educational and work participation, and quality of life for young people with JIA. Importantly, the results may extend beyond JIA and be transferable to other inflammatory diseases in children, such as inflammatory bowel disease, uveitis, psoriasis, and connective tissue diseases using the same combination therapy.

While the study is designed to reflect real-world clinical practice, some limitations should be acknowledged. A potential risk is the challenge of recruiting a sufficient number of eligible participants, particularly given the relatively low prevalence of patients with JIA in sustained remission on combination therapy. To address this, the trial is designed as a multicenter study to maximize reach and ensure a broad and diverse participant pool. If recruitment targets are not met as planned, the primary study center will increase its recruitment efforts, and additional centers may be invited to join the trial. This flexible recruitment strategy is intended to ensure that the study achieves its target sample size and maintains statistical power. Additionally, the generalizability of the results may be influenced by differences in healthcare systems and access to biologic therapies. Also, as with all pragmatic clinical trials, there may be challenges related to treatment adherence and follow-up.

Nevertheless, the MOVE-JIA trial is expected to generate high-quality, clinically relevant data that will form a scientific basis for optimized long-term JIA management and inform future treatment recommendations. By identifying the most effective and well-tolerated approach to maintenance treatment, all outcomes from the trial will provide a scientific basis for optimized JIA care and more evidence-based treatment recommendations.

## Trial status

At the time of manuscript submission, all 7 study sites have started recruitment, and 40 participants are included in the study. The recruitment started in October 2024 and is expected to be complete by the end of 2026.

## Data Availability

Personal data will be stored for 25 years after the end of the study to comply with the requirements in Regulation (EU) No 536/2014 (Clinical Trials Regulation). Data can be made available upon request to the sponsor after anonymization and a signed data sharing agreement.

## Abbreviations

AEs: Adverse events
CRP: C-reactive protein
CTIS: Clinical trial information system
CTU: Clinical Trial Unit
DMARD: Disease modifying anti-rheumatic drug
eCRF: Electronic case report file
ESR: Erythrocyte sedimentation rate
ILAR: International League of Associations for Rheumatology
JADAS: Juvenile Arthritis Disease Activity Score
JIA: Juvenile idiopathic arthritis
OUH: Oslo University Hospital
PROMs: Patient-reported outcome measures
RCT: Randomized controlled study
REK: Regional ethics committee for medical and health research
SAEs: Serious adverse events
SAP: Statistical analysis plan
S.C.: Subcutaneous
SOA: Schedule of activities
SUSAR: Suspected unexpected seri
